# Mutation in SUMO E3 ligase, SIZ1, Disrupts the Mature Female Gametophyte in *Arabidopsis*

**DOI:** 10.1371/journal.pone.0029470

**Published:** 2012-01-09

**Authors:** Yu Ling, Chunyu Zhang, Tong Chen, Huaiqing Hao, Peng Liu, Ray A. Bressan, Paul M. Hasegawa, Jing Bo Jin, Jinxing Lin

**Affiliations:** 1 Key Laboratory of Plant Molecular Physiology, Institute of Botany, Chinese Academy of Sciences, Beijing, China; 2 Graduate School of Chinese Academy of Sciences, Beijing, China; 3 Department of Horticulture and Landscape Architecture, Purdue University, West Lafayette, Indiana, United States of America; 4 Plant Stress Genomics Center, King Abdullah University of Science and Technology, Thuwal, Saudi Arabia; 5 Biological Science Department, King Abdulaziz University, Jeddah, Saudi Arabia; 6 Division of Applied Life Sciences, Gyeongsang National University, Jinju, South Korea; University of Melbourne, Australia

## Abstract

Female gametophyte is the multicellular haploid structure that can produce embryo and endosperm after fertilization, which has become an attractive model system for investigating molecular mechanisms in nuclei migration, cell specification, cell-to-cell communication and many other processes. Previous reports found that the small ubiquitin-like modifier (SUMO) E3 ligase, SIZ1, participated in many processes depending on particular target substrates and suppression of salicylic acid (SA) accumulation. Here, we report that SIZ1 mediates the reproductive process. SIZ1 showed enhanced expression in female organs, but was not detected in the anther or pollen. A defect in the *siz1-2* maternal source resulted in reduced seed-set regardless of high SA concentration within the plant. Moreover, aniline blue staining and scanning electron microscopy revealed that funicular and micropylar pollen tube guidance was arrested in *siz1-2* plants. Some of the embryo sacs of ovules in *siz1-2* were also disrupted quickly after stage FG7. There was no significant affects of the *siz1-2* mutation on expression of genes involved in female gametophyte development- or pollen tube guidance in ovaries. Together, our results suggest that SIZ1 sustains the stability and normal function of the mature female gametophyte which is necessary for pollen tube guidance.

## Introduction

Female gametophyte plays a pivotal role in the sexual reproduction of angiosperms. It is the structure that produces the egg cell and central cell which give rise to the seed embryo and endosperm after fertilization, respectively. In addition, the female gametophyte regulates reproductive processes such as pollen tube guidance, fertilization, and the induction of seed development.

Over recent decades, evidence has accumulated regarding the function of female gametophyte on pollen tube guidance. *Arabidopsis* ovules carrying *magatama3* (*maa3*) [1] or *protein disulfide isomerase like2-1* (*pdil2-1*) [2] disrupt gametophytic pollen tube guidance due to delays in embryo sac maturation, indicating that pollen tube guidance signal(s) emanate only from mature ovules. Elegant cell ablation experiments in *Torenia fournieri* and studies on a synergid-expressed *MYB98* gene in Arabidopsis indicate that synergid cells are the origin of pollen tube guidance signals [3], [4]. This is further supported by findings on pollen tube guidance attractants (LUREs) in *Torenia fournieri* [5]. Studies on synergid- and egg-expressed signal protein ZmEA1 in maize (*Zea mays*) [6], *central cell guidance* (*ccg*) and the *GABA Transaminase* (*pop2*) mutants in Arabidopsis [7] provide evidences that other cells in or surrounding the embryo sac would function in pollen tube guidance. However, no study focus on how does the mature female gametophyte maintain its function.

The small ubiquitin-like modifier (SUMO) E3 ligase, SIZ1, has been described previously as participating in many processes depending on SUMO modification of its substrate proteins. SIZ1 regulates Pi deficiency responses [8] and facilitates basal thermotolerance [9]. Another report showed that SIZ1-dependent SUMOylation of ICE1 may activate and/or stabilize the protein, facilitate activation of C-Repeat (CRT)/dehydration responsive element (DRE) binding protein 1A (CBF3/DREB1A) and repression of *MYB15*, leading to low-temperature tolerance [10]. Recently, SUMOylation of ABI5 by SIZ1 was demonstrated to negatively regulate abscisic acid signaling [11]. SIZ1 suppresses salicylic acid (SA) accumulation and involved in plant innate immunity and cell division and elongation, the expression of *nahG*, a bacterial salicylate hydroxylase that catabolizes SA, in *siz1* plants results in reversal of these phenotypes back to wild-type [12], [13]. Furthermore, Jin and colleagues revealed that SIZ1 negatively regulated transition to flowering under short-days by regulating Flowering Locus D (FLD) and SA-dependent pathways [14]. However, whether and how SIZ1 participates in regulating the plant reproductive process remains unclear.

In the present study, we showed that SIZ1 expressed in the female organs, affected reproductive efficiency during gametogenesis. Some *siz1-2* ovules harbored defective female gametophytes after stage FG7 and disrupted gametophytic pollen tube guidance. Moreover, no significant change was detected in the transcription levels of several previously reported genes required for female gametogenesis or pollen tube guidance between the *siz1-2* ovary and that of the wild type. Based on these findings, the potential roles of SIZ1 in regulating female gametogenesis were discussed.

## Results

### SIZ1 is expressed in reproductive organs

**Figure 1:**
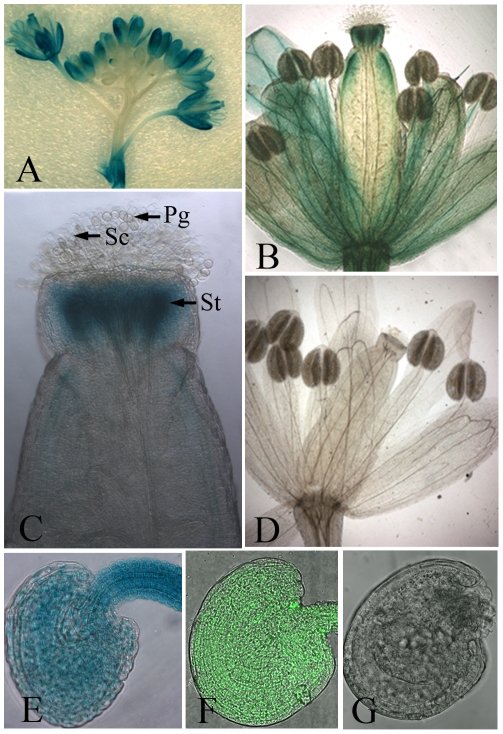
Expression of the Pro_siz1_::GUS–GFP gene. (A) Expression of Pro_siz1_::GUS–GFP in the whole inflorescence. A GUS signal was detected in most of the flowers, except the latest ones, while the strongest GUS signal was found in sepals. The inflorescence in (A) was stained with 1 mM 5-bromo-4-chloro-3-indolyl-b-glucuronic acid (X-Gluc) for 12 h. (B) GUS signal in a flower after pollination. The style of the pistil was stained strongly by X-Gluc, and the upper part of the carpel and the stem of the stamen were also stained by X-Gluc. No GUS signal was seen in the anthers. (C) GUS signal in reproductive organs at the end of pollination. The style was strongly stained by X-Gluc, while no GUS signal was seen in the stigmatic cells or pollen. (D) No GUS signal was detected in the whole flower in the wild-type plants after staining with 1 mM X-Gluc for 12 h. (E) Expression of Pro_siz1_::GUS–GFP could be detected in all cells within the ovule before fertilization after staining with 1 mM X-Gluc for 8 h. (F) GFP fluorescence of Pro_siz1_::GUS–GFP can be seen in all cells of the ovules. (G) Wild-type ovule control. No fluorescence was detected in the wild-type ovule under LSCM. Pg, pollen grain; Sc, stigmatic cell; St, style.

Several lines of single-copy homozygous transgenic plants, which contained an in-frame fusion of a *SIZ1* promoter to a GUS–GFP fusion protein in the Col-0 genetic background, were generated. The SIZ1 promoter used has been demonstrated to be fully functional [14]. As a result, GUS activity was detected in the flowers at different developmental stages, both open and closed, except for those most recently formed (Figure 1A). Apart from the strongest GUS activity detected in the petals, GUS activity was also clearly present in the sepals and pistil, while no obvious GUS activity was seen in the anthers (Figure 1A, B). In terms of the female reproductive organs, GUS expression was particularly high in the style, while a lower level of GUS signal was also detected in other parts of the pistil (Figure 1B, C). Inside the ovary, GUS activity was observed in the whole ovule, including the funiculus (Figure 1E). In contrast, we did not see GUS activity in pollen grains (Figure 1B, C). Furthermore, consistent with the GUS analysis results, GFP signal was present in all the cells within the ovules (Figure 1F). No GUS activity or GFP signal was detected in the controls (Figure 1D, G).

### SIZ1 regulated ovule development

**Figure 2:**
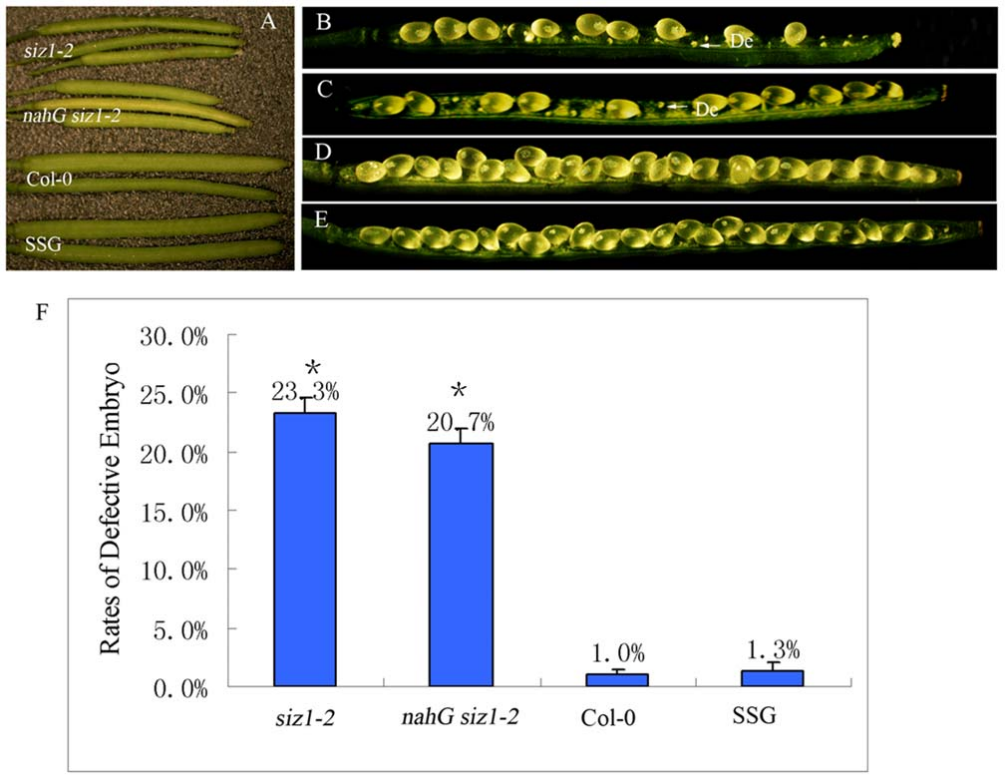
Silique development and seed-set of *siz1-2*, *nahG siz1-2*, wild-type, and the *Pro_siz1_::SIZ1-GFP* construct-transformed *siz1-2* mutant plants (*SSG*). (A) Siliques of *siz1-2*, *nahG siz1-2*, wild-type, and *SSG* 8–10 days after pollination. (B) Dissected silique from *siz1-2* homozygous plants showing severely reduced seed-set and undeveloped ovules. Similar results were also found in line *siz1-3* (data not shown). De, defective embryo. (C) Dissected silique from *nahG siz1-2* plants showing severely reduced seed-set and undeveloped ovules, similar to *siz1-2*. De, defective embryo. (D) Dissected silique of a wild-type plant with a full seed-set. (E) Dissected silique of a *SSG* plant with full seed-set, similar to that of the wild-type plant. (F) Percentage of defective embryos in *siz1-2*, *nahG siz1-2*, Col-0, and *SSG* pistils. A mean value of three repeats, asterisks indicate no significant difference between percentage of defective embryos of *siz1-2* and *nahG siz1-2* (P<0.05).

To determine if SIZ1 regulates reproductive processes, silique size and seed diameter and number were compared between Col-0 and *siz1-2* plants. The *siz1-2* silique was significantly smaller than that of wild type 8–10 days after pollination (DAP; Figure 2A). Moreover, two significantly different populations of seeds were observed in the siliques of *siz1-2*, among which some seeds were well developed (Figure 2B, D). The others harbored desiccated ovules which might stop growing at early stages. Quantitative analysis showed that 23.3% (±1.3%) of the ovules in *siz1-2* siliques were desiccated, but only 1.0% (±0.5%) of ovule was desiccated in the wild-type siliques (Figure 2F). To determine if the desiccated ovule phenotype of the *siz1-2* is due to mutation in the *SIZ1*, we analyzed ovule development in the transgenic plants that expressing *Pro_SIZ1_::SIZ1-GFP* in *siz1-2* (*SSG*), The *SSG* transgenic plants has been confirmed to rescue most of the other *siz1-2* phenotypes, such as early short day flowering phenotype [14]. Consistent with previous results, we also found that expression of SIZ1-GFP could rescue the impaired ovular development phenotype of *siz1-2*. The silique size in *SSG* plants was similar to that of the wild type, and only 1.3% (±0.7%) of defective seeds were found in *SSG* siliques, indicating that the abnormal phenotypes of siliques and seeds in *siz1-2* were caused by the absence of *SIZ1* (Figure 2A, E, F). Several *siz1-2* mutant phenotypes, such as innate immunity, early flowering and cell division and elongation, are associated with elevated SA [12], [13], [14]. To check if the ovule phenotype of the *siz1* is due to elevated SA, we generated *nahG siz1-2* plants by crossing *nahG* transgenic plant and *siz1-2* [13]. *nahG siz1-2* plants has been confirmed accumulate basal level of SA [13], [14]. Notably, reduced SA levels by nahG in *siz1-2* did not rescue the impaired phenotype of ovules during the reproductive process (Figure 2A, C, F). The siliques at 8–10 DAP in *nahG siz1-2* were similar to those of *siz1-2*, which were shorter and smaller than those of wild-type plants (Figure 2A). Moreover, both normal seeds and desiccated ovules were found in the dissected *nahG siz1-2* siliques, and further analysis demonstrated that 20.7% (±1.2%) of the seeds in the dissected *nahG siz1-2* siliques were desiccated, similar to the impaired phenotype of *siz1-2* ovules (Figure 2C, F).

### Absence of SIZ1 did not influence embryogenesis or male gametogenesis

**Figure 3:**
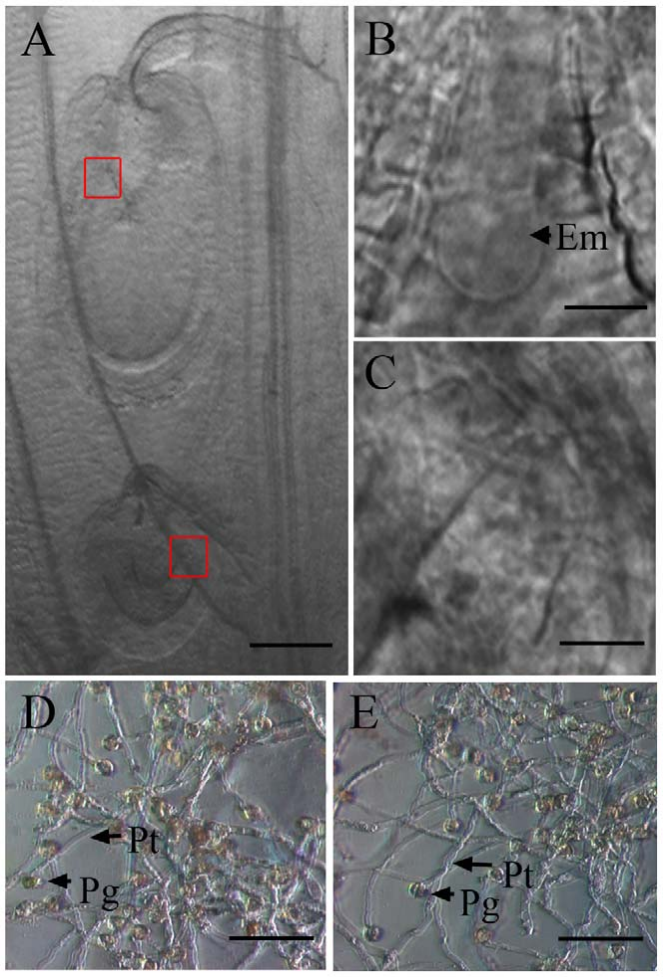
Analysis of ovule development and *in vitro* germination of *siz1-2* pollen grains compared to the wild type by DIC microscopy. (A)–(C) Ovules in a *siz1-2* mutant under a DIC microscope. The fertilized ovule grew bigger and formed a quadrant embryo (Em) within the embryo sac (B); the unfertilized ovule stopped growing, with no proembryo appearing. (D) Wild-type pollen tubes cultured at 28°C *in vitro*. Pollen tubes with normal morphology are indicated by an arrow. (E) *siz1-2* pollen tubes incubated under the same condition as (A), showing no obvious difference compared to the wild-type pollen tube. Em, embryo. Pg, pollen grain. Pt, pollen tube. Bar = 50 µm in (A), 8 µm in (B) and (C), 200 µm in (D) and (E).

Under DIC light microscopy, we found that within the siliques of *siz1-2* at 2 DAP, the fertilized ovules contained well developed proembryos (Figure 3A, B). Nevertheless, some smaller ovules were observed in *siz1-2* siliques, about 100 µm in size, similar to those of the unfertilized ovules. Moreover, no proembryo was detected in these ovules (Figure 3A, C), suggesting that they were not fertilized.

To assess whether the defects in *siz1-2* pollen caused fertilization failure, the function of *siz1-2* and wild-type pollen was examined through in vitro germination assays and by reciprocal pollinations between wild-type and *siz1-2* plants. When pollinated with pollen grains from homozygous *siz1-2* plants, the pistils of wild-type seedlings gave rise to normal siliques with nearly full seed-set 8–10 DAP, more than 99.0% (n = 221) of ovules developed well. In contrast, when pollinated with pollen grains from wild-type seedlings, the pistils in homozygous *siz1-2* seedlings produced shorter and smaller siliques with impaired ovule development, which were similar to those in the self-pollinated homozygous *siz1-2* plants, 21.3% (n = 437) of ovules in these siliques were aborted (Figure S1B). The *in vitro* pollen germination showed a similar pattern to the results from the reciprocal crossing analysis. About 78.6% of pollen grains from *siz1-2* germinated after cultivation on agarose, similar to the germination rate of wild-type pollen (82.2%). Both wild-type and *siz1-2* pollen produced a mass of pollen tubes with similar appearance, and no obvious difference was observed in the maximum length or morphology of wild-type (Figure 3D) and *siz1-2* pollen tubes (Figure 3E). Together, these results indicated that the *siz1-2* male gametophyte functioned normally.

### Some ovules in *siz1* pistils do not attract pollen tubes

**Figure 4:**
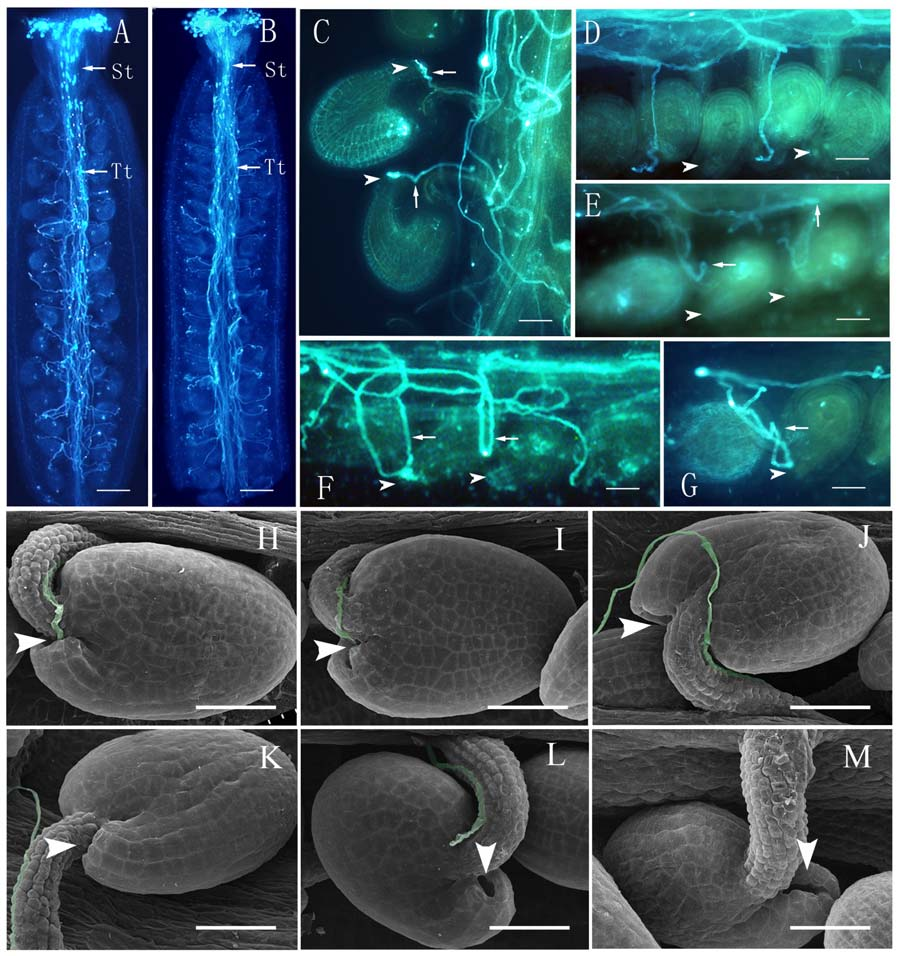
Pollen tube growth in the pistils from wild-type and homozygous *siz1-2* plants. (A)–(G) decolorized aniline blue staining of pistils 1–2 days after pollination (DAP). (A) The whole scene of pollen tube growth within the *siz1-2* pistil. (B) The whole scene of pollen tube growth within the wild-type pistil. (C) The fertilized wild-type ovules showing pollen tubes (arrow) grew into the micropyle (arrowhead) and became bigger in volume. The pistil was harvested 2 days after pollination. (D) Mutant ovules without a pollen tube growing toward the funiculus, while many pollen tubes grew within the placenta. (E) The mutant ovule with pollen tube (arrow) growing around the funiculus, but turning away from the funiculus, without targeting the micropyle (arrowhead). (F) Two undeveloped ovules with pollen tubes (arrow) growing around the funiculus without targeting the micropyle. (G) Representative mutant ovule with pollen tube growing near the micropyle opening but failing to target the female gametophyte. (H)–(M) Scanning electron microscopy analysis of pistils 1–2 days after pollination. (H) Scanning electron micrograph of wild-type ovules showing that pollen tubes grew along the funiculus and then entered the micropyle (arrowhead). (I) Scanning electron micrograph of some *siz1-2* ovules showing that pollen tubes grew along the funiculus and then entered the micropyle (arrowhead), similar to those of the wild type. (J)–(M) Aberrant pollen tube guidance in *siz1-2* ovules. (L) A pollen tube stopped growing near the micropyle (arrowhead). (J) A pollen tube bypassing the micropyle and growing on the surface of the integument. (K) A pollen tube grew along the funiculus but failed to enter the micropyle and turned away. (M) An example showing that no pollen tube grew on the funiculus of the ovule. St, style; Tt, pollen tube transmitting tract. Arrows indicate pollen tubes and arrowheads show micropyle. Bar = 200 µm in (A) and (B), 40 µm in (C)–(M).

Abundant pollen tubes germinated and grew successfully through the stigmatic cell, style, and the pollen tube transmitting tract in the *siz1-2* pistil, similar to those of wild-type plants (Figure 4A, B), indicating that pollen tube growth and sporophytic guidance were normal in *siz1-2* pistils during the sporophytic guidance stage. In contrast, the pollen tube behaved differently in *siz1-2* and wild-type pistils during the gametophytic guidance stage. The pollen tubes in wild-type siliques grew along the funiculus and entered the female gametophyte successfully, after leaving the transmitting tract, 99.2% (n = 267) of ovules received pollen tubes (Figure 4C). In *siz1-2* ovaries, 17.9% of ovules (130 of 724) did not have a pollen tube arriving at the micropylar opening. Some pollen tubes appeared to have lost their way soon after they grew out from the transmitting tract because they did not appear on the funiculus of some ovules (11.3%, n = 724) (Figure 4D, Figure S2). Other pollen tubes could grow on the funicular tissue, they even arrived near the micropylar opening of the ovules (6.6%, n = 724), but turned away and failed to enter the embryo sacs (Figure 4E, F, G, Figure S2).

Using scanning electron microscopy (SEM), pollen tubes could be seen adhering tightly to the funiculus and grow toward the micropyle, precisely entering the micropylar opening of the ovule in the wild-type siliques (Figure 4H). Although pollen tubes were present in most of the *siz1-2* ovules, they behaved differently after presenting from the septum. Most of pollen tubes grew along the funiculus and entered the micropyle of some ovules in *siz1-2* pistils (Figure 4I). However, the pollen tubes in other ovules failed to find the micropylar opening and grew without definite direction (Figure 4J, K, L); some of them bypassed the micropyle and grew on the ovule surface (Figure 4J) or even turned away (Figure 4K), or ceased to grow near the micropyle (Figure 4L). Furthermore, no pollen tube growth was found on the funiculus in a small proportion of *siz1-2* ovules (Figure 4M).

### SIZ1 is required to maintain the stability of the stage FG7 embryo sac

**Figure 5:**
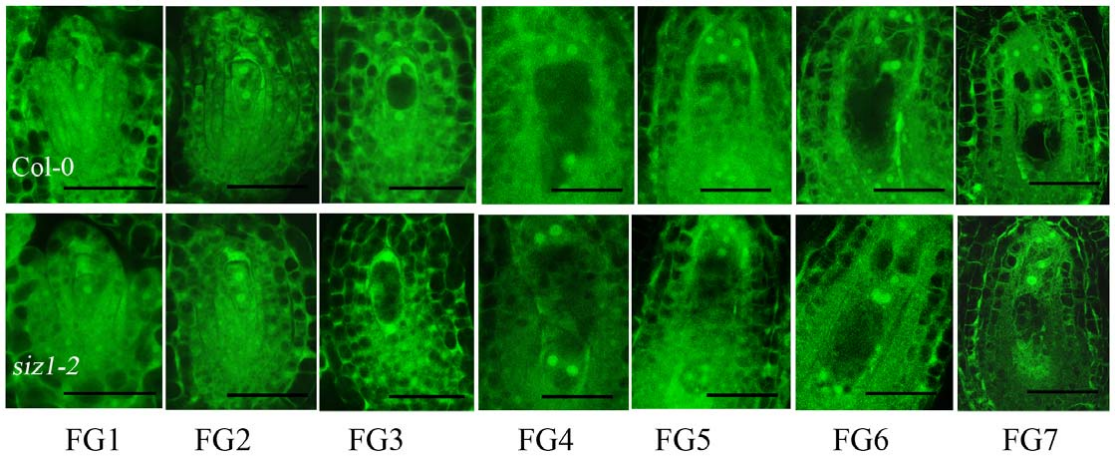
Ovule development from stage FG1 to FG7 in the wild type and *siz1-2* mutant. The upper panels show ovule development of the wild type, as revealed by laser scanning confocal microscope, while the lower panels show ovule development of the *siz1-2* mutant. Corresponding development stages of the ovules examined are indicated below. Bar = 20 µm.

We next asked whether *siz1-2* ovules developed and functioned normally. Thus, pistils at different developmental stages were collected from *siz1-2* and wild-type plants. Confocal microscopy showed no obvious difference in ovule development between *siz1-2* seedlings and the wild-type seedlings before floral stage 12c (as defined by Smyth et al. [15]). Typical embryo sacs of ovules at different developmental stages could be found in *siz1-2* and wild-type pistils (Figure 5). We found that about half of the ovules in each silique at floral stage 12c from the *siz1-2* seedlings were at developmental stage FG7, while the other ovules were at earlier stages, such as FG4, FG5, and FG6; no ovule harbored an abnormal embryo sac, similar to those in wild-type siliques, indicating that *siz1-2* female gametophyte developed normally as far as FG7.

**Figure 6:**
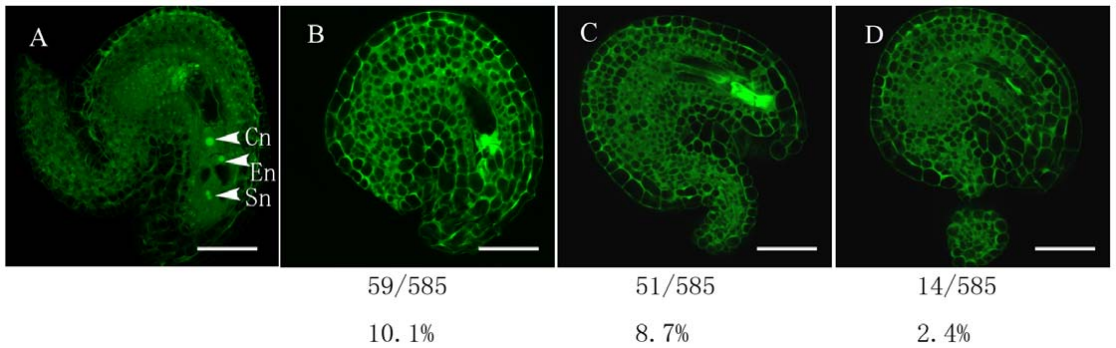
Final phenotypes of the female gametophyte in the wild type and *siz1-2* mutant. (A) LSCM images of an ovule derived from a wild-type flower; the pistil was harvested 2 day after emasculation. (B)–(D) LSCM images for ovules derived from *siz-1-2* flowers; the pistils were harvested 2 days after emasculation. Percentages of abnormal female gametophytes among the examined ovules are indicated below. Cn, central cell nucleus; En, egg cell nucleus; Sn, synergid cell nucleus. Bar = 40 µm.

After stage 12c, we found that many ovules in the *siz1-2* pistils contained normal embryo sacs, which could be fertilized normally, similar to those in wild-type pistils (Figure 6A). However, *siz1-2* pistils contained 21.2% (n = 585 ovules) abnormal ovules, which had normal integument and distorted gametophytic cells within the embryo sac. As shown in Figure 6B, C, D, we did not detect any nucleus within the embryo sac of 10.1% of *siz1-2* ovules, although profiles of gametophytic cells were clearly differentiated (Figure 6B). Additionally, 8.7% of the ovules harbored distorted embryo sacs and the gametophytic cells were permeated with fluorescent blocks (Figure 6C). Shrunken embryo sacs with weak fluorescent blocks were observed in the rest of the ovules (2.4%).

### Expression levels of genes related to female gametophyte development and pollen tube guidance did not change in *siz1-2*

**Figure 7:**
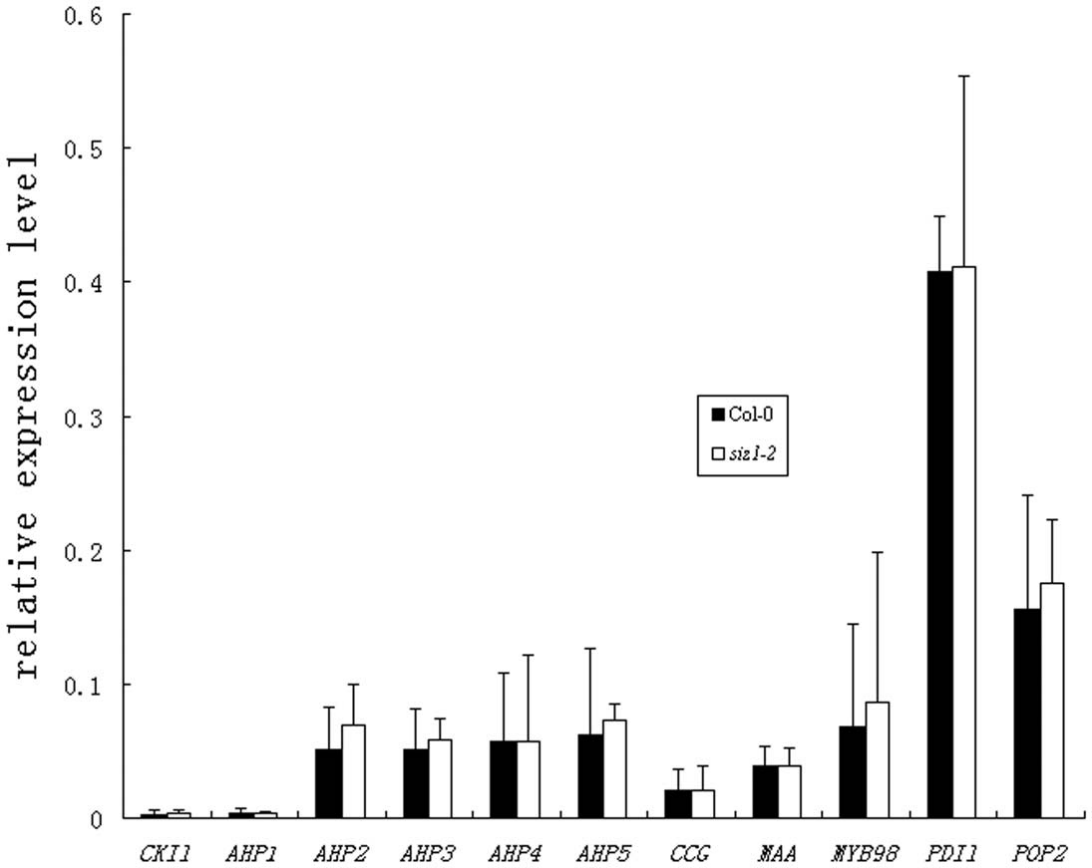
Expression patterns of selected genes from *siz1-2* and wild-type siliques. The tubulin α-2 gene (AT1G04820) was used as the internal control, and its expression level was set arbitrarily as 1.

To explore whether *siz1* mutation affected the expression of previously reported genes related to female gametophyte development or pollen tube guidance, qRT-PCR analyses were performed. cDNAs were prepared from ovary RNA samples of *siz1-2* and wild-type plants at stage 12c. As shown in Figure 7, although the expression levels varied among different genes, the expression level of the genes showed no obvious difference between *siz1-2* and wild-type plants. Loss-of-function in a two-component response system component, CYTOKININ-INDEPENDENT1 (CKI1) and its downstream proteins, HISTIDINE PHOSPHOTRANSFERs (AHPs), containing collapsed embryo sacs, were considered to be disrupted during developmental stage FG5/FG6 in the female gametophytic process of cellularization [16]. The *CKI1* transcript was detected at the lowest abundance and showed no significant difference in its expression level between the wild-type and the *siz1-2* ovaries, and the expression levels of its downstream genes, *AHPs*, in *siz1-2* were similar to those of the wild type, indicating that their expression was apparently unaffected by the absence of SIZ1. Although the expression levels of pollen tube guidance-related genes, *CCG*, *MAA3*, *MYB98*, *PDIl2-1*, and *POP2*, varied in different cDNA samples, their expression levels did not change significantly in *siz1-2* compared to the wild type.

## Discussion

Pollen development is essential for plant reproduction, and some proteins have been shown to be involved in successful fertilization by regulating pollen tube growth in *Arabidopsis*. Jiang and colleagues found that the VANGUARD1 (VGD) was required for polarized growth of the pollen tube, possibly by modifying the cell wall and enhancing the interaction of the pollen tube with the female style and transmitting tract tissues [17]. Recently, a knockout mutation in *THERMOSENSITIVE MALE STERILE 1* (*TMS1*), grown at 30°C, was reported to have greatly retarded pollen tube growth in the transmitting tract, resulting in a significant reduction in male fertility [18]. In the present study, we found that the *siz1-2* mutant produced shorter and small siliques, in which 23.3% (±1.3%) of unfertilized ovules were distributed along septum. We had preliminarily presumed that the defective phenotype during reproductive process in *siz1-2* could be caused by defects in pollen grain germination or pollen tube growth, but the results from reciprocal crossing between homozygous *siz1-2* and wild-type plants and *in vitro* pollen activity analysis showed no significant difference in pollen grain germination and pollen tube development between *siz1-2* and wild-type seedlings. From these observations, we suggest that the absence of SIZ1 does not affect pollen development, and the reduced seed-set in the *siz1-2* mutant may be due to other causes.

The developmental pattern of female gametophytes in most angiosperm species is the Polygonum type, including *Arabidopsis thaliana*, in which a diploid megaspore mother cell undergoes meiosis to produce four haploid megaspores. Among them, one of the megaspores survives and the other three degenerate (FG1), the functional megaspore undergoes the first round of mitosis (FG2), followed by the formation of the central vacuole between the two nuclei (FG3), subsequently the second round of mitosis creates a fournucleate cell (FG4) and the last mitosis produces an eightnucleate cell (FG5). Afterwards, nuclear migration and cellularization result in the seven-celled embryo sac (FG6), and finally the three antipodal cells degrade and the mature embryo sac forms (FG7) [19]. It was found that the female gametophytic division cycle was arrested in *slow walker2* (*swa2*), which led to the growth arrest of the female gametophytes at the two-, four-, or eight-nucleate stage [20]. Pagnussat et al. (2007) revealed that an extra functional egg cell can be detected instead of a synergid in the embryo sac of the *eostre* mutant, which underwent disordered nuclear migration from FG3 [21]. In addition, mutations in *LACHESIS* (*LIS*) and *GAMETOPHYTIC FACTOR1* (*GFA1*) showed disruption after cellularization by changing the cell identities inside the embryo sac [22], [23]. In contrast, the *binding protein1 binding protein2* (*bip1 bip2*) double mutation were defective in the fusion of polar nuclei during their development [24]. The embryo sacs of mutations in *CKI1* or *AHPs* disrupted during developmental stage FG5/FG6, the process of cellularization [16]. In our study, no ovule contained a disrupted embryo sac in *siz1-2* siliques of floral stage 12c, in which about half of the ovules were at developmental stage FG7, similar to those in wild-type siliques, indicating that *siz1-2* female gametophyte developed normally without abnormalities in cell division, nuclear migration, gametophytic cell identities or polar nuclei fusion in embryo sacs before stage FG7. However, 48 h after stage 12c, 21.2% (n = 585) of the ovules contained distorted embryo sacs in *siz1-2* pistils, and the profiles of cellularized female gametophytic cell were still detected within those impaired embryo sacs, suggesting that some part of the mature embryo sacs collapsed rapidly in *siz1-2*. Furthermore, the expression levels of *CKI1* and *AHPs* were unaffected by the absence of SIZ1. These results suggest that SIZ1 has a role in sustaining the stability of the mature embryo sac, rather than being involved in the development and cellularization of female gametophytic cells.

Kinds of proteins in the ovule affect pollen tube guidance by different mechanisms. POP2 regulates both pollen tube growth and guidance by influencing the asymmetric distribution of GABA in the sporophytic cells surrounding the female gametophyte [25]. MYB98 and CCG have been shown to regulate pollen tube guidance via effects on synergids and the central cell, respectively [7], [26]. MAA3 and PDIL2-1 regulate female gametogenesis to provide a normal rhythm of guidance signals [1], [2]. In the present study, we found that some mature ovules in *siz1* mutants failed to attract pollen tubes, while the absence of SIZ1 did not lead to a change in the mRNA levels of those proteins previously identified to be involved in pollen tube guidance, seemingly ruling out the possibility that SIZ1 might work as a transcription regulator for these genes. Based on different traits in the impaired female gametophytes of *siz1* and other mutants defect in female gametophyte development, we conclude that SIZ1 affect pollen tube guidance by sustaining the stability and normal function of mature female gametophyte.

*siz1* mutants have elevated salicylic acid (SA) levels, which could be restored to basal level by expression of the bacterial salicylate hydroxylase gene *nahG* [13]. The mechanism for SA accumulation in *siz1* mutants is not elucidated thus far, which may be attributed to the SP-RING domain of the SIZ1 protein [27]. Several experimental results indicated that by suppressing SA accumulation, SIZ1 played roles in innate immunity [13], and cell division and elongation, control of leave number and volume, and dwarfism [12]. These phenotypes restored to wild type in *nahG siz1-2* plants. In contrast, the impaired phenotypes of female gametophyte development in *nahG siz1-2* plants were similar to those in *siz1-2*, suggesting that the disruption of female gametophyte development was independent with elevated accumulation of SA in *siz1* plants. Given the complex relationship between SA and SIZ1, the results in the present study were insufficient to define the roles of SA at basal levels in female gametophyte development.

SIZ1 has been found to play roles in many different aspects via its SUMO E3 ligase function. Most of the previous reports showed that SIZ1-dependent SUMOylation was involved in many stress process, including responses to Pi deficiency [8], unfavorable temperature [9], [10], flowering time control [14], and abscisic acid signaling regulation [11]. Saracco et al. showed that a *SUMO1* and *SUMO2* double mutant, mutations affecting SUMO-activating enzyme subunit *SAE2* and the SUMO-conjugating enzyme *SCE1* (the only SUMO E2 enzyme in *Arabidopsis*), were embryonic-lethal; fertilized zygotes were aborted at various stages during early embryogenesis [28], indicating that the SUMOylation pathway was essential for embryogenesis. Although these mutants appeared to undergo normal male and female gametophyte development, we can not rule out the possibility that SUMOylation may influence gametogenesis process since low SUMOylation levels can still be detected in these mutants. The present study demonstrated that mature female gametophytes were rapidly disrupted in the absence of the SIZ1 protein, while other ovules survived and developed well, indicating that SIZ1 plays important roles in female gametogenesis.

In our study, only 20∼25% of female gametophytes aborted in *siz1-2* pistils, since both *siz1-2* and *siz1-3* are null mutants [8]. First, presumably in addition to AtSIZ1, there are other SUMO E3 ligase(s) (for example, HPY2/MMS21 [29], [30]) may function redundantly during female gametophyte development. Recently it has been found that diSUMO-like ZmDSUL regulated female gametophyte in maize [31]. In the ZmDSUL-RNAi lines 26% of female gametophytes have not been fully differentiated. AtSIZ1 mainly involved in SUMOylation of SUMO1 and SUMO2 in Arabidopsis [28]. It is interesting to test whether another SUMO E3 ligase(s), such as HPY2/MMS21, facilitates SUMOylation of SUMO3/4/5/6 that regulates female gametophyte in Arabidopsis. Another alternative possibility is that the AtSIZ1 regulates balance of histone methylation status in the genes that are required for female gametogenesis. Female gametogenesis require precise gene regulatory networks [32], [33]. The HMTs (histone methyltransferases) and HDMs (histone demethylases) monitor dynamic histone methylation status, which is required for high order gene expression regulation [34]. In Arabidopsis, a SET-domain protein, SDG2 (SET DOMAIN GROUP2), regulates H3K4 methylation status, is required for gametophyte development [35], [36]. Clough et al. (2007) found that another SET-domain protein, Egg, regulated oogamete development by regulating trimethylation of histone H3K9 in *Drosophila* ovary [37]. MBD1 (methyl-CpG-binding domain1) was found to modulate histone methylation of H3K9 by forming stable or transient complex with a HMT protein, SETDB1 [38], [39]. Recently, Mathhew et al. (2006) revealed that PIAS-mediated SUMOylation of MBD1 inhibited the formation of MBD1/SETDB1 complex, and overexpression of PIAS1 repressed the SETDB1-mediated histone H3K9 methylation of *p53BP2* [40]. As a member of PIAS-family, AtSIZ1 and other PIAS proteins share high sequence identity and show conserved functions [10], [41], [42]. In the future, it is interesting to test whether AtSIZ1 interacts with a SET-domain protein to maintain balance of histone methylation status in Arabidopsis ovules.

## Materials and Methods

### Plant Material and Growth Conditions

Arabidopsis (*Arabidopsis thaliana*) Col-0 ecotype genetic resources for this research were the wild type, *siz1-2* [8], *nahG siz1-2* [9], *Pro_SIZ1_::GUS–GFP* (single-copy homozygous transgenic plants that contained an in-frame fusion of a *SIZ1* promoter to a GUS–GFP fusion protein in the Col-0 genetic background) and *SSG* (*Pro_SIZ1_::SIZ1-GFP*-expressing *siz1-2* plants) [14]. Arabidopsis plants were grown under long-day conditions (16-h-light/8-h-dark) at 22°C.

### Pollen Germination and Microscopy

Pollen from open flowers was suspended in growth medium as described by Palanivelu et al. [25], which contained 18% sucrose, 0.01% boric acid, 2 mM CaCl_2_, 1 mM MgSO_4_). 2–3 µL of the pollen suspension was spotted on growth medium containing 0.5% purified agarose (Bio-Rad). Wild-type and *siz1-2* pollen grains were transferred on the same petri-dish and incubated at 28°C for 16 hr. Images were captured on a Zeiss Axiovert with Axiovision software.

### Fluorescence Staining of Pollen Tubes

To visualize the *in vivo* geminated pollen tubes, siliques were processed as previously described by Huck [43] in a modified method. Siliques were opened with a fine needle under a stereoscope and fixed immediately at room temperature 16 hr in the fixation solution containing 10% acetic acid, and 90% ethanol. The fixed sample was hydrated by passing through an alcohol series (70, 50, 30 and 10%) with 10 min for each step. The sample was further softened with 1 M NaOH at 65°C for 24 hr, subsequently rinsed twice with 100 mM sodium phosphate buffer, pH 7.0, each for 5 min. Pollen tubes were stained with 0.1% aniline blue (Sigma-Aldrich) for 10 min and washed three times with the sodium phosphate buffer before observation. Stained samples were observed using a Zeiss Axioplan microscope (Carl Zeiss) equipped with an epifluorescence UV filter set (excitation filter at 365 nm, dichroic mirror at 395 nm, barrier filter long-pass at 420 nm).

### GUS Assays

GUS staining was performed according to Vielle-Calzada et al. [44]. Pistils and siliques were opened and incubated in GUS staining solution (1 mg/mL X-Gluc [Biosynth], 2 mM K_4_Fe(CN)_6_, 2 mM K_3_Fe(CN)_6_, 10 mM EDTA, 0.1% Triton X-100, and 100 mg/mL chloramphenicol in 50 mM sodium phosphate buffer, pH 7.0) for 2 to 3 d at 37°C. The stained sample was fixed with 70% ethanol. Stained ovules and sections were observed on a Zeiss Axioplan microscope with Nomarski and dark-field optics.

### Pollination Experiment

The stamen at floral stage 12c was emasculated by carefully removing the stamens. After 24 h of emasculation, pollen grains from wild-type or mutant seedlings were dispersed onto the papillar cells of the recipient stigma. Pistils were allowed to set seeds or checked microscopically at different times after pollination for pollen tube entry.

### Seed-Set Analysis

To analyze seed-set, siliques 8 to 10 d after fertilization were placed on double-sided tape and transversely dissected under a stereoscope, and then undeveloped and normal ovules were counted. Images of siliques were taken with a Nikon SMZ800 stereoscope. For reciprocal crosses with Col-0, flowers were emasculated in the morning and crossing was performed 24 h later.

### Laser Scanning Confocal Microscopy

To study the cytological structure of the female gametophyte, ovules were fixed and observed as described previously [19]. Inflorescences were fixed in 4% glutaraldehyde in 12.5 mM cacodylate buffer, pH 6.9, and dehydrated through a conventional ethanol series and subsequently cleared in 2∶1 of benzyl benzoate: benzyl alcohol. Then, siliques were opened with a 30.5-gauge syringe along the replum, and ovules were mounted with immersion oil and sealed under No. 0 cover slips (ProSciTech) with fingernail polish. The developmental stages of ovules were determined according to the criteria described by Christensen et al. [19]. The sample was then viewed with a Zeiss laser scanning microscope (Carl Zeiss Meta 510, Wetzlar, Germany) with a 488-nm argon laser and a long-pass 530 filter. Serial optic sections were collected and projected with Zeiss LSM Image Browser software (Carl Zeiss) and Photoshop version 7.0 software (Adobe).

### Scanning Electron Microscopy

For scanning electron microscopy, pistils from both wild-type and *siz1-2* plants 1 to 2 DAP were carefully opened with a sharp needle and then fixed with FAA (50% ethanol, 3.7% formaldehyde, and 5% acetic acid) overnight. After a series of dehydration steps using increasing concentrations of ethanol of 70%, 80%, 90%, 95% and 100%, the pistils were washed successively in series of the ethanol- to-amyl acetate ratios of 3∶1, 1∶1, 1∶3, and at last 100% amyl acetate in an amyl-acetate-resistant container (10–20 minutes per step). Subsequently the pistils were subjected to critical point drying, then mounted for sputter coating with gold palladium for 100 s and observed on a Hitachi S-4800 scanning electron microscope at an accelerating voltage of 10 kV.

### RNA extraction and preparation

Pistils of 12c were used for analysis. Trizol reagent (Invitrogen, Carlsbad, CA, USA) was used for the RNA extraction, and RNA samples were further treated with DNase to eliminate DNA contamination.

### qRT- PCR analysis

Polymerase chain reactions were performed with an Mx3000P Real-Time PCR System (Stratagene, CA), using SYBR_ Green to monitor dsDNA synthesis. The reaction system was as followed: 10 µL 23 SYBR_ Green Master Mix reagent (TOYOBO CO, OSAKA, JAPAN), 1 µL of 1∶5 diluted reverse transcription reaction, and 400 nM of each gene-specific primer in a final volume of 20 µL. The following standard thermal profile was used for all PCRs: pre-denaturation at 95°C for 1 min; denaturation at 95°C for 5 s, annealing at 58°C for 10 s, and prolongation at 72°C for 15 s, 40 cycles. Data were analyzed using Mx3000P system software (Stratagene, CA).

The data were analyzed using the comparative CT (threshold cycle) method. In order to compare the data from different PCR runs or cDNA samples, CT values for genes were normalized to the CT value of TUB2, which was a housekeeping gene included in each PCR run. The sequences of the primer pairs used were CKIl-f: AGGTCGAACAATGCGACAG; CKIl-a: CTCTCTAGTTGCTTCATAGC; AHP1-f: CCAAGACTCTGATAGGATTC; AHP1-a: GGAAGACAACACAAGCATTC; AHP2-f: GCTCTCATTGCTCAGCTTC; AHP2-a: CTGATAAGCTTCACACAATC; AHP3-f: TTGTGGCTGAGGTTGTTACT; AHP3-a: ACTCCTTGAGGGTAACACAA; AHP4-f: GAAGAGCTCCAAGATGATGC; AHP4-a: TGATGCATGTAACTATCCAG; AHP5-f: TGAAGGGTGTCTAAGGTGTTT; AHP5-a: TTGTGTCATCAGCCTTGAAC; MAA-f: CACTGTTGATGGGTTCCAG; MAA-a: TGAACCAACGACCAATACTG; MYB98-f: AATGGACTGCTGAAGAAGAC; MYB98-a: TCTATCAACACTCTGTCCTC; PDIl-f: GTGGCAGGGATTTAGATGAC; PDIl-a: CTTGCTTCCTCTTCTATGCG; CCG-f: CGAGTTCTTTGCTGGTTTAGA; CCG-a: GTTTCCATCGCTAAATCTGCT; POP2-f: CATTCTTTGGAGCCGAGTG; POP2-a: TGCTGAGCCTTGAGTTCTT; TUB-f: TTTACCCATCTCCACAGGTC; TUB-a: AATAACCTGAGAGACGAGGC.

## Supporting Information

Figure S1 Dissected silique of Col-0 seedling pollinated with *siz1-2* pollen and dissected silique of of *siz1-2* seedling pollinated with Col-0 pollen. (A) Dissected silique from *siz1-2* plants pollinated with Col-0 pollen showing severely reduced seed-set and undeveloped ovules. De, defective embryo. (B) Dissected silique from Col-0 plants with a full seed-set. (C) *siz1-2* pistils pollinated with wild type pollen grains resulted in 21.3 (±3.2)% (n = 437) of aborted ovules, whereas only about 0.9 (±0.1)% (n = 221) of ovules did not fertilize when wild type pistils were pollinated with *siz1-2* pollen grains.
(TIF)

Figure S2 Rates of pollen tubes guidance defect in wild type and *siz1-2* pistils. When the pistils of *siz1-2* plants were pollinated with wild type pollen grains, about 11.3% of ovules (n = 724) did not attract pollen tubes to the funiculus (marked as Fd), and 6.6% of ovules (n = 724) had pollen tubes on the funiculus, but failed to grow into the micropylar opening of the ovules (marked as Md). When wild type pistils were pollinated with wild type pollen grains, only 0.8% of ovules (n = 267) did not have pollen tubes on the funiculus (marked as Fd), other ovules had pollen tubes on the funiculus and they can grow into the micropyle successfully.
(TIF)
