# Supplementary figures and images for: Mutation in SUMO E3 ligase, SIZ1, Disrupts the Mature Female Gametophyte in Arabidopsis
Source: PLoS One. 2012 Jan 9;7(1):e29470. doi: 10.1371/journal.pone.0029470 (PMC3253799; doi:10.1371/journal.pone.0029470)

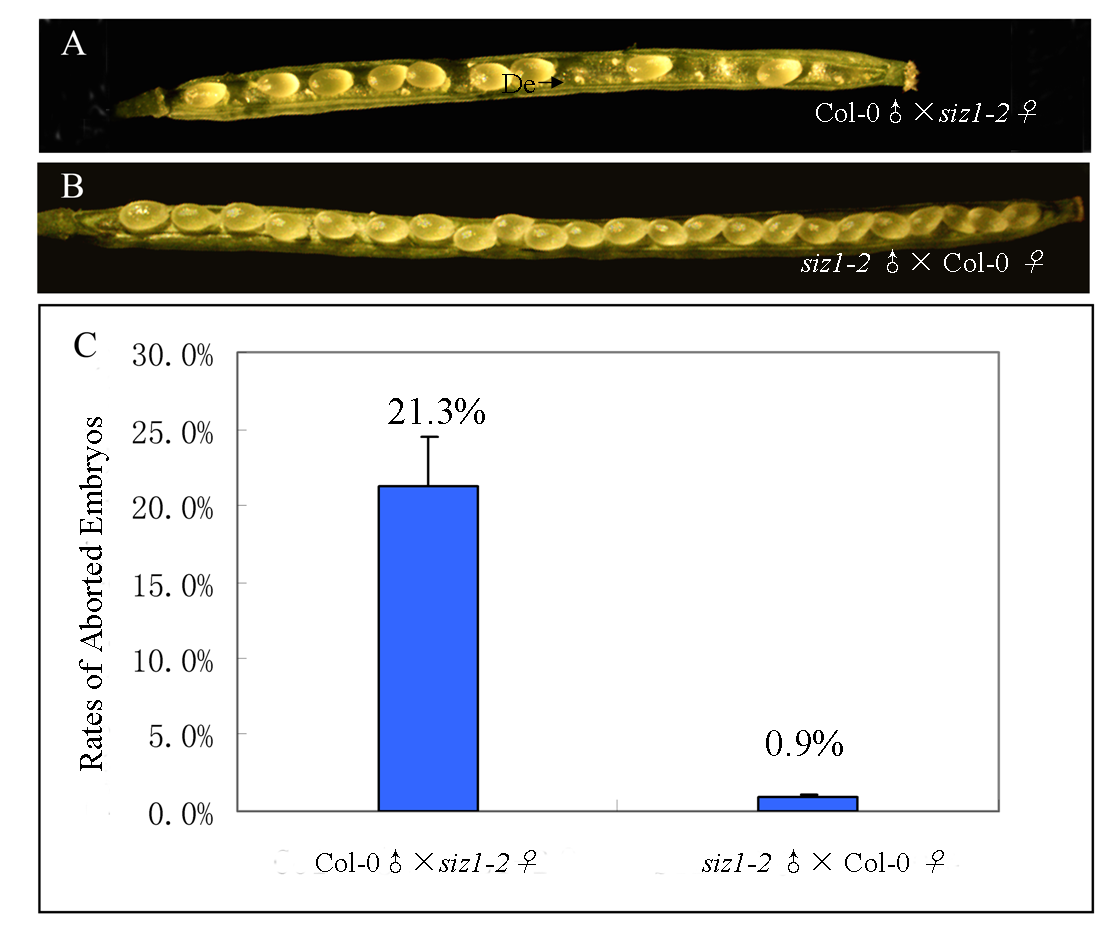

Supplement: Figure S1 — Dissected silique of Col-0 seedling pollinated with siz1-2 pollen and dissected silique of of siz1-2 seedling pollinated with Col-0 pollen. (A) Dissected silique from siz1-2 plants pollinated with Col-0 pollen showing severely reduced seed-set and undeveloped ovules. De, defective embryo. (B) Dissected silique from Col-0 plants with a full seed-set. (C) siz1-2 pistils pollinated with wild type pollen grains resulted in 21.3 (±3.2)% (n = 437) of aborted ovules, whereas only about 0.9 (±0.1)% (n = 221) of ovules did not fertilize when wild type pistils were pollinated with siz1-2 pollen grains. (TIF) [file pone.0029470.s001.tif]

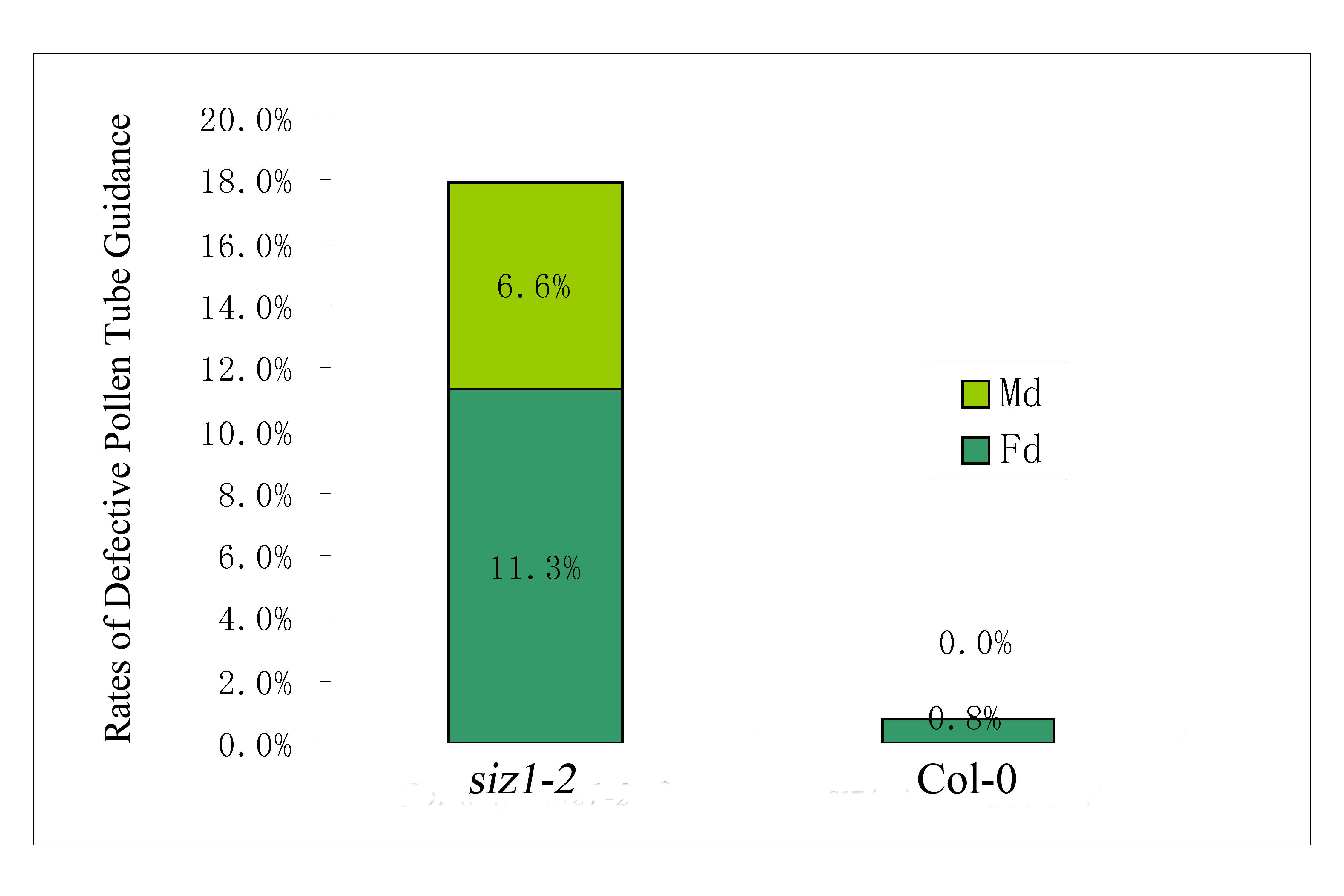

Supplement: Figure S2 — Rates of pollen tubes guidance defect in wild type and siz1-2 pistils. When the pistils of siz1-2 plants were pollinated with wild type pollen grains, about 11.3% of ovules (n = 724) did not attract pollen tubes to the funiculus (marked as Fd), and 6.6% of ovules (n = 724) had pollen tubes on the funiculus, but failed to grow into the micropylar opening of the ovules (marked as Md). When wild type pistils were pollinated with wild type pollen grains, only 0.8% of ovules (n = 267) did not have pollen tubes on the funiculus (marked as Fd), other ovules had pollen tubes on the funiculus and they can grow into the micropyle successfully. (TIF) [file pone.0029470.s002.tif]
